# Intra-rater reliability of hallux flexor strength measures using the Nintendo Wii Balance Board

**DOI:** 10.1186/s13047-015-0104-7

**Published:** 2015-09-09

**Authors:** June Quek, Julia Treleaven, Sandra G. Brauer, Shaun O’Leary, Ross A. Clark

**Affiliations:** University of Queensland, St Lucia, QLD 4072 Australia; Department of Physiotherapy, Singapore General Hospital, Outram Rd, Singapore, 169608 Singapore; Royal Brisbane and Women’s Hospital, Herston, Brisbane, QLD 4029 Australia; School of Exercise Science, Faculty of Health Sciences, Australian Catholic University, Fitzroy, VIC 3065 Australia

**Keywords:** Nintendo Wii Balance Board, Hallux flexor strength, Intrinsic foot muscles, Intra-rater reliability

## Abstract

**Background:**

The purpose of this study was to investigate the intra-rater reliability of a new method in combination with the Nintendo Wii Balance Board (NWBB) to measure the strength of hallux flexor muscle.

**Methods:**

Thirty healthy individuals (age: 34.9 ± 12.9 years, height: 170.4 ± 10.5 cm, weight: 69.3 ± 15.3 kg, female = 15) participated. Repeated testing was completed within 7 days. Participants performed strength testing in sitting using a wooden platform in combination with the NWBB. This new method was set up to selectively recruit an intrinsic muscle of the foot, specifically the flexor hallucis brevis muscle. Statistical analysis was performed using intra-class coefficients and ordinary least product analysis. To estimate measurement error, standard error of measurement (SEM), minimal detectable change (MDC) and percentage error were calculated.

**Results:**

Results indicate excellent intra-rater reliability (ICC = 0.982, CI = 0.96-0.99) with an absence of systematic bias. SEM, MDC and percentage error value were 0.5, 1.4 and 12 % respectively.

**Conclusions:**

This study demonstrates that a new method in combination with the NWBB application is reliable to measure hallux flexor strength and has potential to be used for future research and clinical application.

## Background

Hallux flexor muscle strength is a significant determinant of balance and functional ability in older adults [1] and an independent predictor of falls in this population [2]. Whilst hallux flexor strength is mainly contributed by flexor hallucis longus and flexor hallucis brevis (FHB) muscles, attention is drawn towards FHB because it is one of the intrinsic foot muscles that is thought to be essential for the stability of the longitudinal foot arch [3]. As such training hallux flexor strength has been shown to improve balance [4] and potentially reduce falls in the elderly. However, assessment of intrinsic muscles of the foot with respect to the prevention of falls is largely neglected in clinical and research settings [5]. This is unfortunate as falls in older adults are prevalent [6] and present a substantial health problem for society [7].

One of the challenges encountered by researchers and clinicians is the difficulty in selectively measuring the strength of intrinsic foot muscles. Previous methods to measure the strength of the intrinsic foot muscles such as the paper grip test [8], plantar pressure sensors [9], and various dynamometry methods [10–13] are of questionable validity due to the difficulty in separating intrinsic and extrinsic foot muscle activity during testing [3]. It has been suggested that these methods may promote flexion of the interphalangeal joint during strength tests, a movement that is thought to result from extrinsic foot muscle activity [14]. In a review article, Soysa and colleagues (2012) have recommended the use of hand-held dynamometry (HHD) to measure toe flexor strength as it permits concurrent metatarsophalangeal (MTP) joint flexion and interphalangeal joint extension and thus minimises the contribution of the extrinsic muscles. However, previous study using the HHD did not report any efforts in stabilising the foot or toe to minimise the extrinsic foot muscles contribution [13], thereby questioning the validity of their methodology.

A potentially useful tool which could replicate a HHD assessment of hallux flexor muscle strength is the Nintendo Wii Balance Board (NWBB). The NWBB contains four individual strain-gauge type load cells and is a reliable measurement tool to assess balance [15] and other weightbearing parameters such as asymmetry [15, 16]. Using the NWBB to assess hallux flexor muscle strength is therefore particularly appealing, because if it is shown to be valid it could be used as a centrepiece tool for assessment of multiple components of balance and falls risk. The purpose of this study is to evaluate the use of the NWBB when combined with a purpose built platform for the measurement of hallux flexion strength. Specifically, we assessed the intra-rater reliability of hallux flexor strength measurements using this new method. We hypothesised that this method will provide reproducible measurements of hallux flexion strength as the NWBB/platform configuration minimises extraneous foot movement, thereby improving selectivity of the intrinsic foot muscles [14].

## Methods

### Participants

Thirty healthy individuals (age: 34.9 ± 12.9 years, height: 170.4 ± 10.5 cm, weight: 69.3 ± 15.3 kg, female = 15) were recruited by convenience sampling. Participants were excluded if they reported any foot deformities or ankle problems. All participants gave informed consent as outlined by the institution’s Human Research Committee and all procedures were conducted according to the Declaration of Helsinki.

### Equipment

**Fig. 1:**
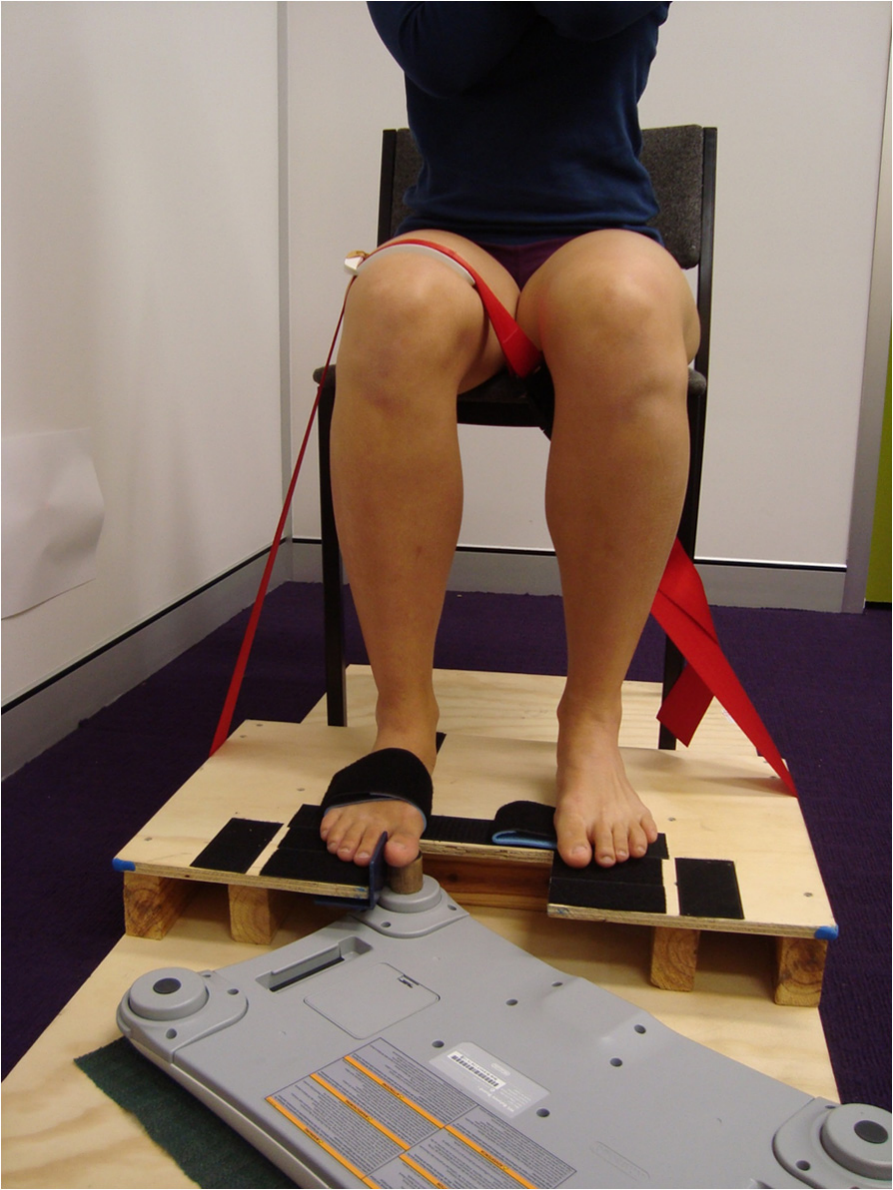
Experimental set up for strength testing of the flexor hallucis muscle of the right foot

For the purpose of this study a NWBB was turned upside down and one of its four load cells was utilised for all measurement procedures (Fig. 1). The NWBB was interfaced via Bluetooth with a customized software program on a Windows computer, and the force data (in kg) was derived using the internally stored calibration information unique to each NWBB. To improve the accuracy of these data, the value derived from the load cell used in the measurement of force was re-calibrated in the testing position. This consisted of applying a range of known loads (0.1–20 kg) to the load cell. A regression equation evaluating the relationship between expected force and that obtained from the NWBB load cell exceeded R^2^ = 0.999 demonstrating the accuracy of the equipment consistent with a previous study [15].

**Fig. 2:**
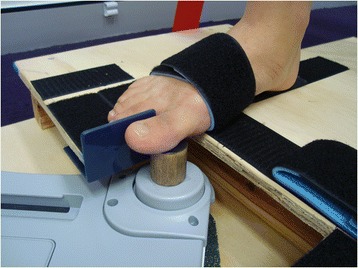
Close up image of the position of the foot on the platform secured with Velcro and the position of the sensor placed under the interphalangeal joint of the big toe

To ensure accurate positioning of the hallux onto the load cell, and to minimise the influence of the extrinsic muscles of the foot (ankle plantarflexors and flexor hallucis longus) and the 2nd to 4th toes, a purpose-built wooden platform was constructed and positioned under the NWBB. As shown in Fig. 2 this permitted the tested foot to be positioned such that the proximal metatarsophalangeal joint of the big toe was at the edge of the platform, and a Velcro strap was used to secure the anterior section of the foot onto the platform. To prevent the heel from lifting adhesive Velcro tape was applied under the participant’s heel and fixed onto Velcro fastened to the platform and the participants thigh was additionally strapped to the platform with a non-elastic belt (Fig. 1). Excessive adduction moments from the big toe were minimized by a toe separator made of smooth plastic that was positioned between the first and second toes and fastened to the platform (Fig. 2). Finally, a circular wooden block of 2.8 cm in diameter was placed over the NWBB load cell to ensure the application point of the load cell was level with the platform and therefore the hallux positioned in its anatomic neutral position for testing.

### Procedure

To assess intra-rater reliability all participants attended two measurement sessions spaced 1–7 days apart. All procedures on both days were identical and supervised by the same physiotherapist (JQ) who has postgraduate qualifications and 13 years of clinical experience in the musculoskeletal field. Participants were seated on a firm chair positioned on the wooden platform (See Fig. 1). The sitting position was standardised using high density foam mats if required, (0 to 4 mats, 1.2 cm in depth each) and positioned on the chair to ensure 90° of hip and knee flexion [17]. The dominant foot was then positioned accurately on the platform and the other foot also rested on the platform.

Participants were given one warm-up trial where they were instructed to apply 50 % of their perceived maximal effort. Participants then performed a minimum of three trials at maximal effort with a 1-min rest period between trials to minimize muscle fatigue. Participants were blinded to the results throughout the testing procedure. Standardised instructions were given throughout testing to; (i) keep the heel planted on the platform, (ii) avoid leaning backwards or forwards at the trunk (iii) avoid flexion at the interphalangeal joint of the big toe and (iv) apply a vertical downward force at the interphalangeal joint of the big toe. To minimize movement from the foot, a Velcro strap was firmly secured across the foot and fixed onto the platform between the first metatarsophalangeal joint to the middle of the arch (see Fig. 2).

To prevent the heel from lifting, besides giving standard instructions, the heel was fixed using a strap placed over the knee. This was also assisted by the fact that the Velcro under the heel would make a sound if the participant raised the heel (indicating use of the calf muscles) during testing. The examiner was alerted to this incorrect manoeuvre by the sound of the Velcro that held the heel to the platform separating. In the event of this occurring the trial would be discarded and repeated. Testing was ceased when three consistent measures were obtained (no greater than 20 % difference in measures) or a maximum of 6 trials had been performed. The average of three consistent measures was calculated. Strength was measured in kilograms.

### Sample size

We determined the sample size of 30 participants using the method proposed by Walter et al. [18]. This was calculated based on a minimum acceptable reliability of 0.70 and an expected ICC value of 0.90 in a test-retest (k = 2) design, with a level (1-tailed) of 0.05 and power of 95 %.

### Statistical analyses

Visual inspection for bias and heteroscedasticity was performed by examining a generated Bland-Altman plot for the difference between the scores obtained on both days against their means. Intra-rater reliability was calculated using intra-class coefficients (ICC [3,3]) and Ordinary Least Product (OLP) regression analysis. To calculate ICC, two-way analysis of variance based on absolute agreement was performed. ICC values of >0.75 were considered excellent, 0.40 to 0.75 modest or <0.40 poor [19]. Next, systematic biases (proportional and fixed biases) were determined using OLP regression analysis [20]. To estimate measurement error, standard error of measurement (SEM), 95 % limits of agreement (LoA), and minimal detectable change (MDC) calculations were performed.

## Results

**Fig. 3:**
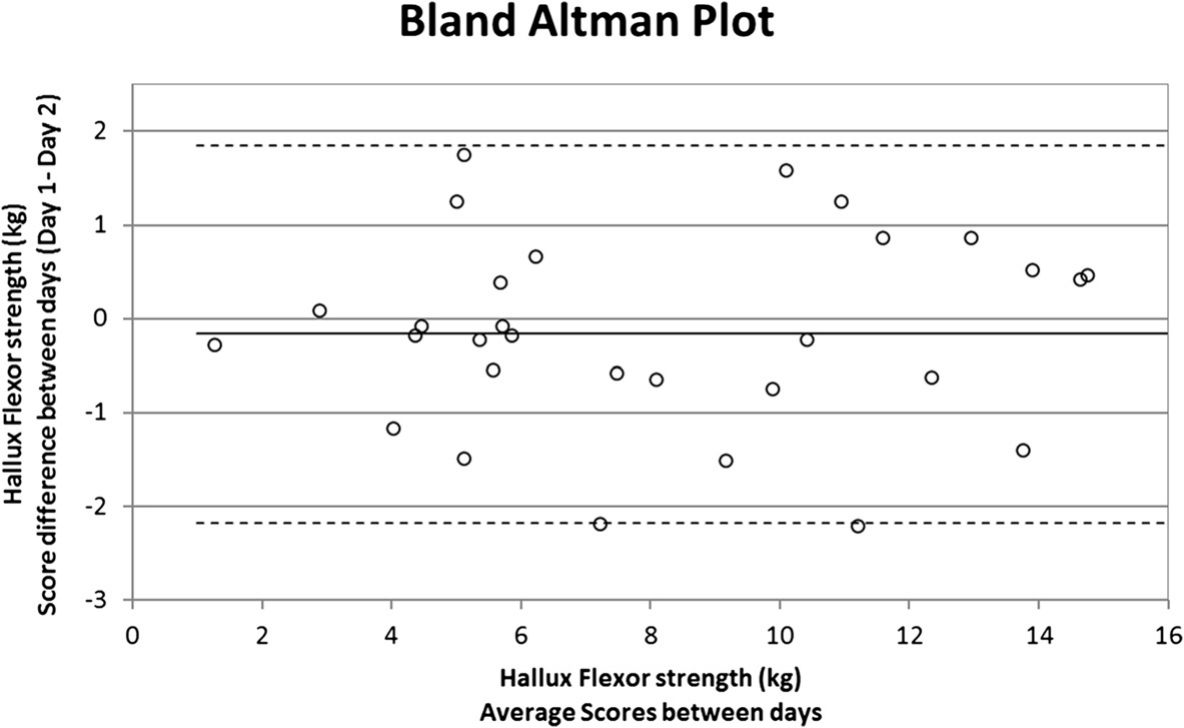
Agreement analysis of measurements between days by Bland Altman plot

The results are presented in Table 1. This new method of hallux flexor strength measurement demonstrated excellent intra-rater reliability (ICC = 0.982, 95 % CI = 0.96 to 0.99) with percentage error of 12 % and no presence of systematic bias and no evidence of heteroscedasticity. The LOA plot is presented in Fig. 3.

**Table 1:** Mean strength recordings (± SD) and reliability coefficients for hallux flexor muscle strength measurement

	Wii board day 1 (Mean ± SD)	Wii board day 2 (Mean ± SD)	ICC (3, 3)	Spearman’s ρ*	95 % CI	Systematic bias (CI)	Width of 95 % LoA	% Error^*b*^	SEM	MDC	Prop bias^a,c ^	Fixed bias^c^
Toe strength (kg)	8.12 ± 3.86	8.28 ± 3.80	0.982	0.933	0.96 to 0.99	None	2.01	12	0.5	1.4	No	No

*SD* standard deviation, *ICC* intra-class coefficients, *LoA* limits of agreement, *SEM* standard error of measurement, *MDC* minimal detectable change

^a^Prop Bias = Proportional Bias

^b^% Error = 0.5*Width of 95 % LOA/[(MeanDay1 + MeanDay2)/2]*100

*All correlations were *p* < 0.001

^c^Prop and fixed bias were determined from ordinary least products analysis

## Discussion

In this study a new method of measuring hallux flexion strength with an emphasis to selectively measure flexor hallucis brevis (an intrinsic foot muscle) strength using the NWBB was described and intra-rater reliability of this method was investigated. The findings suggest this new method reliably measures hallux flexion strength (ICC = 0.982). Moreover the method is inexpensive and straight-forward to set up and therefore has application for research and clinical settings. Furthermore, the NWBB has other useful applications such as for balance assessment [15] and rehabilitation [21], and therefore has diverse clinical and research utility.

To address issues of previous studies [3] the method described in this study attempted to minimise compensatory strategies from the trunk and ankle, as well as extrinsic muscles of the foot, from confounding accurate measurement of the intrinsic foot muscle strength. Specifically we attempted to minimise extrinsic foot muscle activity by localising toe flexion at the MTP joint and extension at the interphalangeal joint [3]. This resulted in force being applied to the load cell under the interphalangeal joint. Strength trials were subsequently ceased and discarded if toe curling was observed. Performing the test in this manner is proposed to optimise selective activity of the FHB, an intrinsic foot muscle, with minimal contribution from the extrinsic foot muscles [14], although future electromyographic studies are required to validate this proposition.

From a clinical perspective this new method permits an accurate and selective measure of flexor hallucis brevis muscle performance, and could have positive implications for clinical assessment and rehabilitation. This is particularly relevant in light of evidence suggesting that improved intrinsic foot muscle strength is associated with improved dynamic support of the medial longitudinal arch and balance [22]. From a statistical perspective, it is useful to note that the percentage error of 12 % would suggest that any differences observed in flexor hallux strength following an intervention must exceed 12 % in order to indicate that a real change has occurred.

### Limitations

This study has some limitations. Firstly we did not assess the inter-rater reliability of the method which potentially limits the applicability between observers. Secondly, although all effort was taken to ensure that participants did not use compensatory strategies by using appropriate physical restraints and standardised instructions, it was not possible to know if the experimental set-up was able to separate the extrinsic and intrinsic foot muscle activity, which will need to be investigated in future studies. Similarly, while we attempted to minimise adduction of the big toe by using a smooth plastic separator between the first and second toes, it was not possible to separate the influence of toe adduction moments during measurements.

### Future considerations

Given that the purpose of this study was to test the reliability of within-subject measures across 2 time-points, we did not need to include variables such as foot arch height and length, the size of interphalangeal joint prominence, the length of the first MTP shaft and the range of motion of MTP joint. However these are important when comparing between subjects, and should be considered in future studies.

## Conclusions

Despite the growing evidence of the importance of maintaining hallux flexion strength for optimal gait and balance in older adults [1, 23], it remains poorly addressed in rehabilitation [5]. The results of this study demonstrate that hallux flexion strength can be reliably assessed using a NWBB application. Given that hallux flexion strength may be an indicator of intrinsic foot muscle performance, this method utilising the NWBB is of great potential for future research and clinical application. Potentially this method may also have application for other foot conditions requiring accurate measurement of hallux flexion strength.

## Abbreviations

FHB: Flexor hallucis brevis
HHD: Hand-held dynamometry
MTP: Metatarsophalangeal
NWBB: Nintendo Wii Balance Board
ICC: Intra-class coefficient
SEM: Standard error of measurement
LOA: Limits of agreement
MDC: Minimal detectable change

## References

[1] Spink MJ, Fotoohabadi MR, Wee E, Hill KD, Lord SR, Menz HB (2011). Foot and ankle strength, range of motion, posture, and deformity are associated with balance and functional ability in older adults. Arch Phys Med Rehabil.

[2] Mickle KJ, Munro BJ, Lord SR, Menz HB, Steele JR (2009). ISB Clinical Biomechanics Award 2009: toe weakness and deformity increase the risk of falls in older people. Clin Biomech (Bristol, Avon).

[3] Soysa A, Hiller C, Refshauge K, Burns J (2012). Importance and challenges of measuring intrinsic foot muscle strength. J Foot Ankle Res.

[4] Kobayashi R, Hosoda M, Minematsu A, Sasaki H, Maejima H, Tanaka S (1999). Effects of toe grasp training for the aged on spontaneous postural sway. J Phys Ther Sci.

[5] McKeon PO, Hertel J, Bramble D, Davis I (2014). The foot core system: a new paradigm for understanding intrinsic foot muscle function. Br J Sports Med.

[6] Hausdorff JM, Rios DA, Edelberg HK (2001). Gait variability and fall risk in community-living older adults: a 1-year prospective study. Arch Phys Med Rehabil.

[7] Lajoie Y, Gallagher S (2004). Predicting falls within the elderly community: comparison of postural sway, reaction time, the Berg balance scale and the Activities-specific Balance Confidence (ABC) scale for comparing fallers and non-fallers. Arch Gerontol Geriatr.

[8] Menz HB, Morris ME, Lord SR (2006). Foot and ankle risk factors for falls in older people: a prospective study. J Gerontol A Biol Sci Med Sci.

[9] Menz HB, Zammit GV, Munteanu SE, Scott G (2006). Plantarflexion strength of the toes: age and gender differences and evaluation of a clinical screening test. Foot Ankle Int.

[10] Unger CL, Wooden MJ (2000). Effect of foot intrinsic muscle strength training on jump performance. J Strength Cond Res.

[11] Senda M, Takahara Y, Yagata Y, Yamamoto K, Nagashima H, Tukiyama H (1999). Measurement of the muscle power of the toes in female marathon runners using a toe dynamometer. Acta Med Okayama.

[12] Kwon O, Tuttle L, Johnson J, Mueller M (2009). Muscle imbalance and reduced ankle joint motion in people with hammer toe deformity. Clin Biomech.

[13] Spink MJ, Fotoohabadi MR, Menz HB (2009). Foot and ankle strength assessment using hand-held dynamometry: reliability and age-related differences. Gerontology.

[14] Garth WP, Miller ST (1989). Evaluation of claw toe deformity, weakness of the foot intrinsics, and posteromedial shin pain. Am J Sports Med.

[15] Clark RA, Bryant AL, Pua Y, McCrory P, Bennell K, Hunt M (2010). Validity and reliability of the Nintendo Wii Balance Board for assessment of standing balance. Gait Posture.

[16] Clark RA, McGough R, Paterson K (2011). Reliability of an inexpensive and portable dynamic weight bearing asymmetry assessment system incorporating dual Nintendo Wii Balance Boards. Gait Posture.

[17] Soma M, Murata S, Kai Y, Nakae H, Satou Y (2014). An examination of limb position for measuring Toe-grip strength. J Phys Ther Sci.

[18] Walter S, Eliasziw M, Donner A (1998). Sample size and optimal designs for reliability studies. Stat Med.

[19] Fleiss JL (1986). The design and analysis of clinical experiments.

[20] Ludbrook J (2002). Statistical techniques for comparing measurers and methods of measurement: a critical review. Clin Exp Pharmacol Physiol.

[21] Gil-Gómez J-A, Lloréns R, Alcañiz M, Colomer C (2011). Effectiveness of a Wii balance board-based system (eBaViR) for balance rehabilitation: a pilot randomized clinical trial in patients with acquired brain injury. J Neuroeng Rehabil.

[22] Mulligan EP, Cook PG (2013). Effect of plantar intrinsic muscle training on medial longitudinal arch morphology and dynamic function. Man Ther.

[23] Misu S, Doi T, Asai T, Sawa R, Tsutsumimoto K, Nakakubo S (2014). Association between toe flexor strength and spatiotemporal gait parameters in community-dwelling older people. J Neuroeng Rehabil.

